# cgMLST typing reproducibility is maintained across diverse whole-genome sequencing workflows in a multi-country HERA2 surveillance pilot

**DOI:** 10.3389/fmicb.2026.1930807

**Published:** 2026-10-28

**Authors:** Adriana Cabal Rosel, Achilleas P. Galanopoulos, Michalis Polemis, Judit Henczkó, Olga Pappa, Eleni Karligiotou, Maria Malliarou, Dimosthenis Chochlakis, Patrick Hyden, Beatriz Daza Prieto, Anna Stöger, Daniel Polzer, Erika Ungvári, Ákos Tóth, Werner Ruppitsch, Dragan Juric, Ivana Ferencak, Irena Tabain

**Affiliations:** 1 Institute for Surveillance and Infectious Disease Epidemiology, Austrian Agency for Health and Food Safety, Vienna, Austria; 2 Institute for Medical Microbiology and Hygiene, Austrian Agency for Health and Food Safety, Vienna, Austria; 3 Laboratory of Hygiene and Epidemiology, Faculty of Medicine, University of Thessaly, Larisa, Greece; 4 Regional Public Health Laboratory (PEDY) of Thessaly, Larissa, Greece; 5 Department of Molecular Epidemiology, Central Public Health Laboratory, National Public Health Organization, Athens, Greece; 6 Division of Microbiological Reference Laboratories, National Center for Public Health and Pharmacy, Budapest, Hungary; 7 Regional Laboratory of Public Health of Crete, School of Medicine, University of Crete, Heraklion, Greece; 8 Department of Statistics and Analytical Epidemiology, Austrian Agency for Health and Food Safety, Vienna, Austria; 9 Institute for Veterinary Disease Control Mödling, Austrian Agency for Health and Food Safety, Mödling, Austria; 10 Institute of Hygiene and Medical Microbiology, Medical University Innsbruck, Innsbruck, Austria; 11 Croatian Institute of Public Health, Zagreb, Croatia

**Keywords:** public health, reproducibility, ring trial, surveillance, whole genome sequencing, cgMLST, ring-trial

## Abstract

**Introduction:**

Whole-genome sequencing (WGS) has become indispensable in infectious disease surveillance, yet its implementation across laboratories remains heterogeneous. Six laboratories from four European countries participated in 2023 in a pilot ring trial as part of the HERA2 project, using a standardized panel of 25 bacterial isolates representing five public health-relevant species. The aim was to assess the comparability and reproducibility of laboratory-specific WGS workflows and to determine whether workflow-related variability affected cgMLST-based interpretations relevant for routine surveillance and outbreak investigations.

**Methods:**

Participating laboratories applied their established wet-laboratory workflows, including different sample-processing, DNA-extraction and library-preparation methods and sequencing platforms. Illumina sequencing was performed by all laboratories, while Oxford Nanopore Technologies (ONT) sequencing was available in two. To specifically evaluate variability introduced by wet-laboratory procedures, all raw sequencing data were centrally processed using the same bioinformatic pipeline. Reproducibility was primarily assessed using cgMLST allelic distances to the Lab 1 Illumina comparator assembly and concordance of cluster assignments, complemented by analyses of sequencing and assembly variability.

**Results:**

Despite substantial differences in wet-laboratory workflows and technical sequencing characteristics, retained Illumina records showed only small cgMLST allelic differences across laboratories (0-2 alleles) and concordant cluster assignments. Sequencing and assembly metrics varied according to laboratory, species, and technical factors, but this variability did not prevent concordant Illumina-based cluster interpretation among records meeting predefined minimum quality requirements. ONT results were more species-dependent, and several records, particularly for Salmonella enterica, did not achieve the required cgMLST target completeness. These ONT findings should be interpreted in the context of the technology available at the time of the 2023 ring trial.

**Conclusions:**

Our results suggest that complete standardization of wet-laboratory workflows may not be required to achieve comparable Illumina-based cgMLST results across laboratories. Rather, laboratories may retain validated local workflows provided that the resulting sequencing data meet appropriate minimum quality requirements. This supports a pragmatic approach to multi-laboratory genomic surveillance that prioritizes data quality and fitness-for-purpose over uniformity of individual wet-laboratory procedures.

## 1 Introduction

Whole-genome sequencing (WGS) has become a transformative tool for public health surveillance, enabling the early detection of outbreaks even when no epidemiological signals are apparent (Struelens et al., 2024). Across the European Union and European Economic Area (EU/EEA), the adoption of WGS for infectious disease surveillance and outbreak investigation expanded rapidly since the mid-2010s. An European Center for Disease Prevention and Control (ECDC) monitoring showed that by 2015, 22 countries already had access to WGS for public health applications, a number that increased to 25 countries in 2016, with roughly half of EU/EEA states using WGS as a first- or second-line typing method for priority pathogens (Revez et al., 2017). Between 2017 and 2019, the vast majority of national reference laboratories were equipped to use WGS for surveillance of diverse pathogens, with the strongest adoption seen for *Listeria monocytogenes* and *Neisseria meningitidis* (ECDC, 2018).

Despite its demonstrated utility and rapid technological development, the large-scale integration of routine WGS into national surveillance systems has been hindered by several constraints. These include high implementation and operational costs, limited laboratory and bioinformatics infrastructures, and shortages of adequately trained personnel (Gomes et al., 2025). Furthermore, the lack of standardized wet lab and dry lab protocols across laboratories can lead to variability in sequence data quality, although such differences do not necessarily compromise the comparability of typing results required for outbreak detection and cluster investigation (Forth et al., 2023).

To address these challenges, and in direct response to the SARS-CoV-2 pandemic, the European Commission launched major capacity- building efforts under the Health Emergency Preparedness and Response Authority (HERA), funded through the EU4Health Programme and the legal framework established by Regulation (EU) 2021/522. In 2021, Member States were supported in expanding and upgrading national infrastructures for both whole-genome sequencing (WGS) and reverse transcription polymerase chain reaction (RT-PCR), thereby enhancing early detection and improving response capacity for pandemics and other cross-border health threats (Välikangas et al., 2022). In 2023, the second wave of HERA actions aimed to reinforce national public health systems by consolidating these enhanced capacities and ensuring their long-term integration into routine genomic surveillance workflows. A central priority was to guarantee the sustainability of the WGS and RT-PCR infrastructures established under the initial HERA actions, extending their application beyond SARS-CoV-2 to additional pathogens of public health relevance.

In 2021, Member States were supported in expanding and upgrading national infrastructures for both whole-genome sequencing (WGS), a technology that determines the complete genetic code of pathogens to track their spread and evolution, and reverse transcription polymerase chain reaction (RT-PCR), the standard method for detecting viral RNA, thereby enhancing early detection and improving response capacity for pandemics and other cross-border health threats

In 2025, the European Commission established a major regulatory milestone by mandating the use of WGS for outbreak investigations involving Salmonella *enterica, Listeria monocytogenes, Escherichia coli, Campylobacter jejuni*, and *Campylobacter coli*. Under Implementing Regulation (EU) 2025/179, all Member States must perform WGS on non-clinical isolates suspected to be linked to foodborne outbreaks and submit the sequences to the European Food Safety Authority (EFSA), with full enforcement beginning 23 August 2026 (Guerra et al., 2025). This regulation marks the transition from uneven national implementation to a harmonized, EU-wide obligation for genomics-based outbreak response within the One Health WGS system jointly developed by EFSA and the ECDC (Guerra et al., 2025). Complementing these regulatory developments, ECDC has developed strategic frameworks and digital infrastructures that support the implementation of WGS for public health surveillance and facilitate genomic data sharing, joint analyses, and interoperability across Member States.

One of the beneficiaries of the second HERA call is the HER*A2* project consortium, comprising Austria, Croatia, Greece, and Hungary. These countries entered the project with varying levels of WGS implementation for surveillance and outbreak investigation, and with differing pathogen specific priorities. Therefore, Work Package 5 (“Implementation and harmonization of alert systems and workflows”) focused on systematically mapping and evaluating existing laboratory capacities and outbreak investigation procedures across participating countries. A pilot laboratory study in the form of a WGS ring trial was conducted. The objective was to evaluate the comparability and reproducibility of laboratory-specific WGS workflows across participating countries and to assess whether workflow-related variability could influence genomic interpretations relevant for routine surveillance and outbreak investigations. To this end, participating laboratories were instructed to sequence the same panel of isolates using their own routine wet-lab workflows, with Illumina sequencing as the primary platform and Oxford Nanopore Technologies (ONT) where available. This design allowed us to assess reproducibility under real-world conditions and to identify potential limitations associated with different sequencing platforms. Here, we describe and compare the results generated by each participating laboratory and evaluate their consistency for epidemiological interpretation.

## 2 Materials and methods

### 2.1 Study set up

A total of six laboratories from four countries (Austria, Croatia, Hungary, Greece) part of the HERA2 project participated in the pilot test. Austria distributed to each participating country a standardized panel of 25 bacterial isolates representing five species of public health importance but also matching the priorities of the countries in terms of pathogen relevance. Pathogens were selected due to either their relevance in multi-country foodborne outbreaks, their role in travel-associated infections or the increasing antimicrobial resistance and included five species such as *Campylobacter jejuni, Salmonella* spp., *Listeria monocytogenes, Legionella pneumophila* and *Enterococcus faecium*. Five isolates per species were included and were obtained from the National Reference Center for Salmonella, Listeria, Legionella and Campylobacter in Austria. *Enterococcus faecium* cultures were obtained from the Institute for Medical Microbiology and Hygiene in Vienna and belonged to a previous project. Each sequenced isolate received a different identification number that started with the pathogen initials followed by HERA23 and the isolate number (e.g.,: Cam-HERA23-16). Materials were shipped on 06.07.2023 from the organizer lab in Austria (Institute of Medical Microbiology and Hygiene, Austrian Agency for Health and Food Safety, Vienna) to the other three countries. Lab3 distributed the materials among its other two regional laboratories.

Each country received two boxes on the same delivery, one containing 15 E-transwabs with Amies liquid and 10 charcoal swabs (Copan Diagnostics, Inc., Corona, CA). Upon arrival to the respective laboratories, an electronic notification via email was sent to the organizers and the arrival date registered in the ring trial protocol form (Supplementary material). Together with the parcels, a protocol with instructions for recording the processed samples and a workbook for recording the sequencing results were sent.

Briefly, laboratories were instructed to sequence at least with Illumina and also with Oxford Nanopore Technologies (ONT) when available. For the pre-analytical part, labs could use the method of their choice, including sample cultivation, DNA extraction, library preparation, and sequencing.

### 2.2 Sample processing and sequencing

#### 2.2.1 Lab 1

Lab 1 was the organizer. The 25 bacterial isolates were recultivated on Columbia agar (BioMérieux, Marcy l'Etoile, France) for 18 h at 37 °C. For Illumina sequencing, DNA was extracted using either the MagAttract HMW kit (Qiagen, Hilden, Germany) or the Mag-Bind Bacterial DNA Kit (Omega Bio-Tek, Norcross, GA, USA), depending on the availability of reagents. For Nanopore sequencing, DNA extraction was performed with the NucleoSpin Microbial DNA kit ((Macherey-Nagel, Düren, Germany). All procedures followed the manufacturers' instructions. Quality control consisted of fluorometric DNA quantification (ng/μl) using the dsDNA HS Assay and Qubit Flex (Thermo Fisher Scientific Inc., Vienna, Austria). Both short- and long-read sequencing were carried out. Illumina libraries were prepared with Nextera XT (Illumina, San Diego, CA, USA), except for *Listeria monocytogenes*, for which the Illumina DNA Prep kit was used. Paired-end sequencing (2 × 150 bp) was performed on a NextSeq 2000 system using a P1 cartridge, targeting a minimum mean genome-wide sequencing coverage of 30 × .

Nanopore libraries were prepared with the Rapid Barcoding Kit SQK RBK114.24 and sequenced on R10.1.4 SpotON flow cells (Oxford Nanopore Technologies, Oxford, UK). Sequencing was conducted on a GridION device for 72 h, followed by SUP basecalling with Guppy v6.3.8.

#### 2.2.2 Lab 2

Upon arrival, isolates were cultivated under species-specific conditions: *Salmonella* spp. on Xylose Lysine Deoxycholate (XLD) agar (Condalab, Madrid, Spain) for 18 h at 35 °C; *Listeria monocytogenes* and *Enterococcus spp*. on blood agar (Liofilchem, Roseto degli Abruzzi, Italy) for 24 h at 35 °C; *Campylobacter spp*. on Campy agar (prepared in-house and supplemented with cefoperazone, rifampicin, and clotrimazole) for 48 h at 42 °C; and *Legionella pneumophila* on BCYE cysteine agar (Oxoid, Basingstoke, UK) for 24 h at 35 °C. Genomic DNA was extracted manually using the NucleoSpin Tissue kit (Macherey Nagel, Düren, Germany) following the manufacturer's protocol, including a 3 h proteinase K digestion at 56 °C. DNA quality was assessed using a 4,200 TapeStation system (Agilent Technologies, Santa Clara, CA, USA), and DNA concentration was determined using Qubit (Thermo Fisher Scientific Inc., Vienna, Austria). WGS libraries (2 × 150 bp) were prepared with the Illumina DNA Prep kit (Illumina, San Diego, CA, USA) and sequenced on a NextSeq 550 platform.

#### 2.2.3 Lab 3

Upon arrival, isolates were stored at 4 °C for up to 3 days. *Salmonella* spp. were cultivated on MacConkey and nutrient agar (OXOID, UK) for 24 h at 37 °C; *Listeria spp*. on ALOA and TSA (OXOID, UK) for 24–48 h at 37 °C; *Campylobacter spp*. on Charcoal Cefoperazone Deoxycholate Agar (CCDA, OXOID, UK) and blood agar for 48 h at 41.5 °C; *Enterococcus spp*. on blood agar for 24 h at 35 °C; and *Legionella spp*. on BCYE and nutrient agar for 96 h at 37 °C (OXOID, UK).

Lab3, distributed isolates to the other two regional laboratories within the same country (Lab 4 and Lab 5).

DNA extraction was performed automatically using the MGI Nucleic Acid Extraction Kit (VDR03P 32) (MGI TECH, Germany). Quality control included spectrophotometric A260/280 measurements and fluorometric quantification with Qubit for all five species. Short read sequencing for samples HERA-23-01 to HERA-23-20 was performed using the Illumina Nextera XT library kit on a NextSeq 2000 system with a P1 cartridge (Illumina, San Diego, CA, USA). Samples HERA 23 21 to HERA 23 25 were sequenced with the same library kit on a MiSeq instrument using a MiSeq v2 kit (Illumina, San Diego, CA, USA).

#### 2.2.4 Lab 4

The isolates were not recultivated upon their initial arrival because they were forwarded from Lab 3 to Lab 4 18 days later and were stored at 4 °C until processing. During cultivation, *Salmonella* spp. were grown on XLD agar (Company) for 18–24 h at 37 °C; *Listeria* spp. on ALOA (Company) for 48 h at 37 °C; *Campylobacter* spp. on COS agar plates (Company) for 48 h at both 37 and 42 °C under microaerophilic conditions; *Enterococcus* spp. On COS agar for 18–24 h at 37 °C; and *Legionella* spp. On Buffered Charcoal Yeast Extract (BCYE) for 5 days at 37 °C. DNA extraction was carried out using the Apex KingFisher system (Thermo Fisher Scientific, Inc, Country). Quality control included spectrophotometric absorbance ratios and Qubit quantification, with concentrations ranging from 10.8 to 278.9 ng/μl and A260/280 ratios between 1.35 and 2.11. Short read sequencing was performed using the Illumina Nextera XT kit (Illumina, San Diego, CA, USA) on a MiSeq instrument with a MiSeq v2 300 cycle cartridge.

#### 2.2.5 Lab 5

Isolates were not recultivated upon their initial arrival because they were transferred from Lab3 to Lab 5 18 days later and were stored at −80 °C until processing. During cultivation, *Salmonella* spp. were grown on XLD (Oxoid, UK) and BGA (Oxoid, UK) for 24 h at 36 °C; *Listeria* spp. on ALOA (Bioprepare, Greece) and Palcam (Bioprepare, Greece) for 36 h at 37 °C; *Campylobacter* spp. on Campygel plates (Bioprepare, Greece) for 24 h at either 37 or 42 °C; *Enterococcus* spp. on SB agar (Bioprepare, Greece) for 48 h at 36 °C; and *Legionella* spp. on BCYE (Bioprepare, Greece) for 48 h at 36 °C. DNA extraction was performed manually using the Qiagen Blood & Tissue kit (Qiagen, Hilden, Germany). Quality control included A260/280 measurements for all species except Campylobacter, and Qubit quantification for all five species, with concentrations ranging from 0.806 to 25.1 ng/μl and A260/280 ratios around 1.8. Short read sequencing was performed using the Illumina Nextera XT library kit (Illumina, San Diego, CA, USA) on a NextSeq 2000 (2 × 150 bp, P1 cartridge).

#### 2.2.6 Lab 6

Isolate processing began immediately upon arrival. Isolates were plated on sheep blood agar (prepared in-house, National Center for Public Health and Pharmacy, Hungary) and incubated for 18 h at 35 °C. *Campylobacter* spp. isolates were additionally plated on Charcoal Cefoperozone Deoxycholate agar (CCDA, prepared in-house, National Center for Public Health and Pharmacy, Hungary) agar incubated for 48 h at 4235 °C. DNA extraction was carried out automatically using the QIAcube with the DNeasy UltraClean Microbial kit (Qiagen, Hilden, Germany). DNA quality was assessed with Qubit (Thermo Fisher Scientific, Grand Island, NY, USA). Short read sequencing was performed using Illumina DNA Prep (Illumina, San Diego, CA, USA) libraries on a NextSeq 550 system (Illumina, San Diego, CA, USA). Nanopore libraries were prepared with the Rapid Barcoding Kit SQK RBK114.24 (Oxford Nanopore Technologies Ltd., Oxford, United Kingdom) and sequenced on R10.1.4 SpotON flow cells (Oxford Nanopore Technologies Ltd., Oxford, United Kingdom). Sequencing was conducted on a GridION device for 72 h, followed by SUP basecalling with Guppy v6.3.8.

### 2.3 Joint sequence analysis

As this pilot test was a wet-lab exercise aimed at generating high-quality FASTQ files, the organizing laboratory (Lab 1) centrally performed all downstream bioinformatic analyses, including FASTQ quality control, trimming, assembly, and typing, using a single analytical workflow to assess inter-laboratory concordance while avoiding variability introduced by heterogeneous bioinformatic pipelines.

Illumina raw reads were processed with MBioSEQ *Ridom Typer* v9.0.3 (Ridom GmbH). This involved quality trimming (Q20, sliding window 4 bp) and adapter removal followed by de novo assembly with SPAdes v3.11.1. Contigs were filtered to retain those with ≥5 × coverage and ≥200 bp in length. Oxford Nanopore Technologies (ONT) reads were filtered with Filtlong v0.2.1, assembled using Flye v2.9.2 b1786, and polished with Medaka (medaka_consensus v1.8.0, model r1041_e82_260bps_sup_g632). Several assembly quality metrics were assessed, including sequencing depth, assembly size, number of contigs, N50, and cgMLST target recovery. Potential interspecies contamination was assessed using Mash Screen.

Subsequently, assemblies were characterized using classical MLST and cgMLST in MBioSEQ *Ridom Typer*. Isolate relatedness was assessed using the validated cgMLST schemes available at the Ridom nomenclature server and interpreted according to the corresponding official cluster definitions. The following schemes were applied: *Listeria monocytogenes* (1,701 core genes; cluster threshold ≤ 7 allelic differences), *Salmonella enterica* (3,002 core genes; cluster threshold ≤ 10 allelic differences), *Campylobacter jejuni* (637 core genes; cluster threshold ≤ 4 allelic differences), *Enterococcus faecium* (1,423 core genes; cluster threshold ≤ 20 allelic differences), and *Legionella pneumophila* (1,521 core genes; cluster threshold ≤ 10 allelic differences). The same software version was used for all analyses. Cluster assignments and inter-laboratory comparisons were based exclusively on cgMLST allelic profiles; no reference-based SNP mapping or SNP-calling analyses were performed. Hybrid assemblies were not generated, as the goal was to evaluate whether ONT alone could serve as a suitable option to detect genomic clusters.

All Illumina and ONT sequencing data produced in this study have been submitted to the Sequence Read Archive (SRA) and are publicly available under the BioProject accession number PRJNA1484536.

### 2.4 Data processing and filtering

The final dataset comprised one sequencing record (i.e., one dataset entry) per unique combination of biological isolate, laboratory, workflow, and sequencing technology, together with the corresponding sequencing, assembly, typing, and quality-control data. Each of the six participating laboratories was expected to sequence the same panel of 25 biological isolates using Illumina, while Labs 1 and 6 additionally sequenced the panel using ONT, resulting totally in 200 expected sequencing records (150 Illumina and 50 ONT). The same biological isolate could therefore be represented by multiple records across laboratories or sequencing technologies. For each isolate, the assembly generated from the Lab 1 Illumina data was used as the common comparator for cgMLST allelic distance calculations across participating laboratories.

All statistical analyses were performed in R (R Foundation for Statistical Computing, Vienna, Austria), with the packages and functions used for individual analyses specified below.

Analyses were conducted on the full dataset and separately on the Illumina and ONT subsets. Categorical variables considered in the statistical analyses included species, laboratory, sequencing technology, library preparation method, DNA extraction mode, assembler partner, and sequencer. Continuous variables used for multivariate analyses included assembly coverage, assembly size, contig count, N50, cgMLST percentage of good targets, read count, and Q30 base percentage. Percentage symbols and thousands separators were removed before numeric conversion. Median imputation was restricted to PCA-based analyses requiring complete numeric matrices; direct reproducibility analyses and mixed-effects models used available values for the relevant endpoint.

A two-stage filtering approach was applied. First, records were eligible for analysis only if library preparation information was available, assembled coverage was ≥30 ×, and cgMLST good targets were ≥95%; records for which no isolate was recovered were also excluded. These minimum inclusion criteria defined the analysis dataset and were applied before all statistical, reproducibility, and comparative analyses, including cgMLST allelic distance analyses. Records not meeting these criteria were retained for transparency but were not included in downstream analyses.

Second, the robustness of the results to additional quality filtering was evaluated using three strategies: (i) Q30/contig filtering, (ii) PCA-based multivariate outlier filtering, and (iii) combined filtering applying both (i) and (ii). For Q30/contig filtering, records with Q30 base percentage ≥50% and contig count ≤ 500 were retained. For PCA-based filtering, PCA was performed on scaled numeric variables and Mahalanobis distance was calculated using PC1–PC7; records exceeding the χ^2^ threshold at α = 0.01, with degrees of freedom equal to the number of PCs, were classified as multivariate outliers. Combined filtering (iii) was used for all analyses and plots presented in this manuscript.

### 2.5 Univariate statistical analyses

Univariate differences among laboratories were assessed within each species using Kruskal–Wallis tests, with *P*-values adjusted across species using the Benjamini–Hochberg (BH) method. Results were visualized using species-stratified boxplots created through the ggplot2 R package. For all the analyses a 5% significance level was set.

### 2.6 Multivariate statistical analyses

Principal component analysis (PCA) was performed using the *prcomp()* function from the stats R package, with centering and scaling. Assembly size, contig count, and N50 were log1p-transformed prior to PCA to reduce skewness, and variables with near-zero variance or only missing values were excluded. Group differences were assessed using PERMANOVA implemented with the *adonis2()* function from the *vegan* R package, with 999 permutations and marginal testing (by = “margin”). Effect sizes were reported as R^2^ values. Analyses were performed independently for each factor in the full dataset and in the Illumina and ONT subsets, using both unfiltered and filtered datasets. Where applicable, pairwise comparisons were performed with Benjamini–Hochberg correction. Homogeneity of multivariate dispersion was assessed using the *betadisper()* function from the *vegan* R package, followed by permutation testing with *permutest()* using 999 permutations. For filtered datasets, multivariable PERMANOVA models included predictors with at least two levels, using complete cases across the included predictors. Models were not fitted when fewer than two varying predictors remained.

A sensitivity analysis was performed to assess how filtering affected the marginal R^2^ estimates from multifactor PERMANOVA of sequencing and assembly metrics. Analyses were repeated separately for the pooled dataset and the Illumina and ONT subsets under four conditions: no additional filtering, Q30/contig-count filtering, PCA-based outlier filtering, and combined filtering. All conditions were applied after the predefined minimum inclusion criteria. Combined filtering applied the Q30/contig-count criteria first, followed by PCA-based outlier detection within the retained records. For each dataset and filtering condition, the multivariate analysis was repeated and marginal *R*^2^ values were extracted for each estimable explanatory factor. These values were displayed as line plots across filtering conditions to describe the sensitivity of the estimated effect sizes to record exclusion.

### 2.7 Reproducibility analyses

To directly assess cross-laboratory reproducibility, allelic distance to the Lab 1 Illumina comparator was analyzed as the primary typing endpoint. All reproducibility analyses were performed at the biological-isolate level, accounting for repeated measurements across laboratories and sequencing technologies.

Intraclass correlation coefficients (ICCs) for allelic distance were estimated using mixed-effects models with biological isolate and laboratory as random effects. The ICC was calculated as the proportion of total allelic-distance variance attributable to biological isolate identity, with higher values indicating greater consistency of isolate-specific allelic-distance patterns across laboratories.

Bland-Altman analyses were performed for pairwise laboratory comparisons of allelic distance using biological isolates shared between each laboratory pair. Mean differences and limits of agreement were calculated, with the median used when multiple values were available for the same biological isolate-laboratory combination. Coefficients of variation were calculated by laboratory for Q30 base percentage, N50, and contig count to summarize variability in key sequencing and assembly metrics.

### 2.8 Mixed-effects variance partitioning

To estimate the relative contribution of isolate, laboratory, sequencing, and workflow factors to variation in sequencing and assembly metrics, random-intercept mixed-effects models were fitted using *lmer()* from the *lme4* R package. Candidate random effects included biological isolate, laboratory, sequencing technology, library preparation, assembler partner, DNA extraction mode, species, and sequencer. Count-like response variables were log1p-transformed where appropriate, and variance components were expressed as percentages of total variance. Singular fits were retained but interpreted cautiously because they indicate zero or near-zero variance estimates for one or more random effects.

## 3 Results

### 3.1 Sequencing characteristics and laboratory performance

A summary of the sequencing and laboratory characteristics is provided in Table 1. Overall isolate recovery was high among all participants with some exceptions (Supplementary Table 1). Three isolates (Cam-HERA23-16, Lm-HERA23-06, and Lp-HERA23-24) failed to grow in Lab 2 and one isolate (Cam-HERA23-20) did grow but the FASTQ file did not pass the quality control and was excluded. In Lab 6, isolates Cam-HERA23-16 and Cam-HERA23-20 could not be recovered. One isolate from Lab 6 (Lp-HERA23-24) that was sequenced with Nanopore was excluded from the analysis because the corresponding FASTQ file was corrupted and the original could not be recovered.

**Table 1:** Sequencing and laboratory characteristics summary.

Laboratory	Isolates obtained (n)	Storage	DNA extraction mode	DNA extraction kit	Library preparation method	Technology	Sequencer
Lab1	25	immediate processing	manual	MagAttract Qiagen	Illumina DNA Prep, Rapid Barcoding, Nextera XT, Native Barcoding	Illumina, ONT	Nextseq2000, GridION
Lab2	21	immediate processing	manual	Nucleospin	Illumina DNA Prep	Illumina	NextSeq 500/550
Lab3	25	4 °C	automated	MGI Easy Nucleic Acid	Nextera XT	Illumina	Nextseq2000, Miseq
Lab4	25	4 °C	automated	MagMAX Thermofisher	Nextera XT	Illumina	Miseq
Lab5	25	frozen	manual	Qiagen Blood & Tissue	Nextera XT	Illumina	Nextseq2000
Lab6	23	immediate processing	automated	DNeasy UltraClean Microbial kit	Illumina DNA Prep, Native Barcoding	Illumina, ONT	NextSeq 500/550, Gridion

Labs 2 and 6 processed the samples immediately upon arrival, whereas Labs 3 and 4 stored them at 4 °C until processing. Lab 5 kept samples at −80 °C prior to processing. Sample handling was also heterogeneous in terms of workflow: Labs 1, 2, and 5 performed manual processing, while Labs 3, 4, and 6 relied on automated procedures. Four laboratories, namely Labs 1, 3, 4, and 6, used bead-based DNA extraction kits, whereas Labs 2 and 5 employed column-based kits. None of the participating laboratories used the same extraction kit.

Sequencing platforms also varied across laboratories. Labs 1, 3 (isolates 1–20), and 5 used the NextSeq 2000, while Lab 3 (isolates 21–25) and Lab 4 used the MiSeq. Labs 2 and 6 performed sequencing on the NextSeq 500/550. Library preparation methods differed by organism and laboratory. Labs 1 (for *L. pneumophila, S. enterica, E. faecium*), 3, 4, and 5 used Nextera XT, whereas Lab 1 (for *C. jejuni* and *L. monocytogenes*), as well as Labs 2 and 6, used Illumina DNA Prep. For ONT sequencing, Labs 1 (for *L. monocytogenes* and *S. enterica*) and Lab 6 employed Native Barcoding, and Lab 1 additionally used Rapid Barcoding for *L. pneumophila* and *C. jejuni*.

Of the 200 expected sequencing records, data were generated for 144 of 150 Illumina records and 47 of 50 ONT records. After applying the predefined minimum inclusion criteria, 162 records were retained for downstream analyses. Fifteen records had assembled coverage < 30 ×, 33 had < 95% cgMLST good targets, and seven lacked library preparation information. Records could fail more than one criterion; therefore, these categories were not mutually exclusive.

For all but two *L. pneumophila* isolates sequenced with Illumina by Labs 2 and 3, sequence type (ST) was successfully determined. In those two isolates, the *mompS* gene was split across two contigs, preventing its detection. ST could not be determined for all ONT *S. enterica* records from Lab 6, two ONT *S. enterica* records from Lab 1, two ONT *L. pneumophila* records from Lab 6, and two ONT *C. jejuni* records from Lab 1. These isolates had coverage >70 × and contig counts of 1-4 but showed < 95% cgMLST good-target recovery.

### 3.2 Univariate analysis results through sequencing and assembly quality metrics

#### 3.2.1 Q30 base percentage

Q30 base percentage showed highly significant laboratory-associated differences within all species (BH-adjusted *p* ≤ 0.001 for all five species). Clear laboratory-specific patterns were observed as depicted in (Figure 1). Across species, Q30 variability appeared strongly influenced by laboratory and sequencer combination, suggesting that sequencing quality metrics are sensitive to technical implementation.

**Figure 1:**
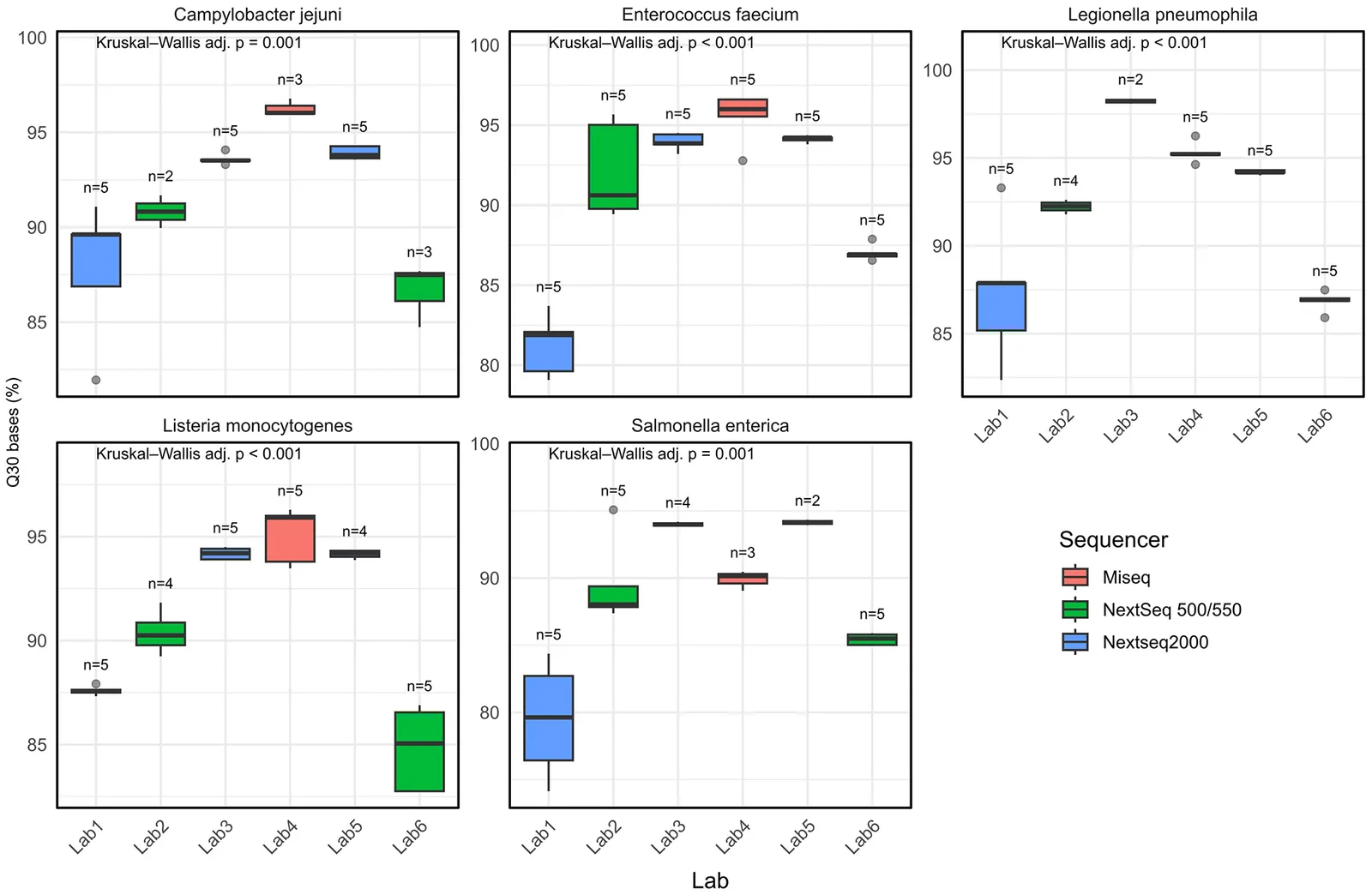
Q30 base percentage across laboratories in the Illumina subset after combined filtering. Boxplots show Q30 base percentages by laboratory, with separate panels for each bacterial species and colors indicating sequencers. Labels indicate the number of sequencing records per group and Benjamini–Hochberg-adjusted Kruskal–Wallis *p*-values for comparisons across laboratories.

#### 3.2.2 Contig count

In contrast to Q30, contig count showed species-dependent laboratory effects. Salmonella enterica demonstrated a statistically significant laboratory effect (BH-adjusted *p* = 0.034), with pronounced variability driven by specific laboratory–sequencer combinations, including extreme dispersion in certain groups. *E. faecium* showed borderline significance (*p* = 0.053). *C*. *jejuni, L*. *pneumophila*, and *L*. *monocytogenes* did not show statistically significant laboratory-associated differences (*p* > 0.05) (Figure 2).

**Figure 2:**
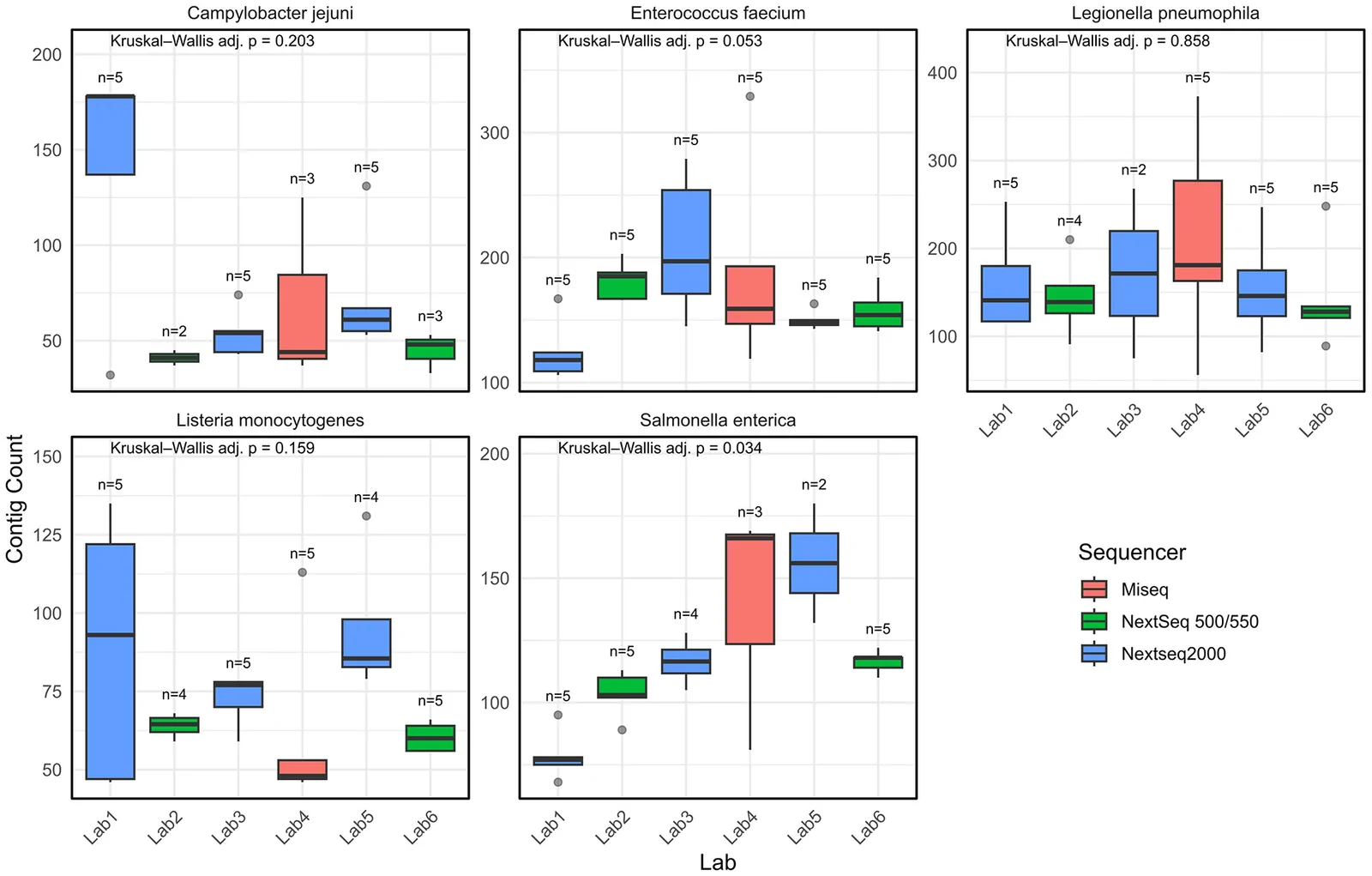
Contig count across laboratories in the Illumina subset after combined filtering. Boxplots show contig counts by laboratory and bacterial species, with colors indicating sequencing instrument. Labels indicate the number of sequencing records per group and Benjamini–Hochberg-adjusted Kruskal–Wallis p-values for comparisons across laboratories.

#### 3.2.3 N50

N50 values showed moderate laboratory-associated variability, but no significant laboratory effects were observed for any species. Inter-species differences were substantially larger than within-species laboratory variation (Figure 3). The *p*-values for comparisons across laboratories were 0.095 for *Enterococcus faecium*, 0.153 for *Salmonella enterica*, 0.148 for *Campylobacter jejuni*, 0.565 for *Legionella pneumophila*, and 0.565 for *Listeria monocytogenes*.

**Figure 3:**
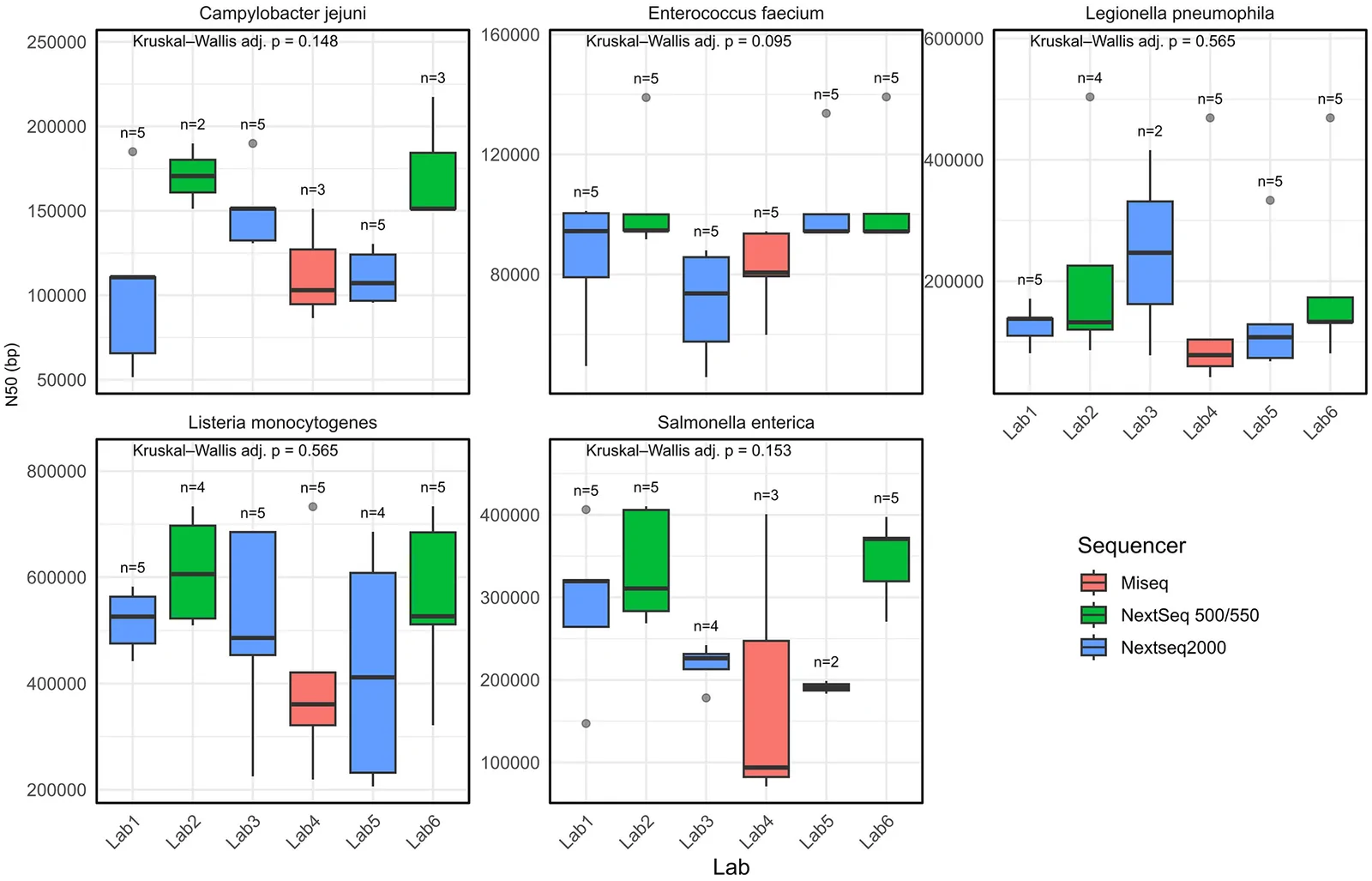
Assembly contiguity across laboratories in the Illumina subset after combined filtering. Boxplots show N50 values by laboratory, with separate panels for each bacterial species and colors indicating sequencing instrument. Labels indicate the number of sequencing records per group and Benjamini–Hochberg-adjusted Kruskal–Wallis p-values for comparisons across laboratories.

### 3.3 Multivariate analysis interpretation

Principal component analysis of the combined-filtered pooled dataset revealed species-associated patterns, with partial separation and overlap among species (Figure 4). The annotated projection also showed separation between Illumina- and ONT-derived sequencing records along PC1. PC1 and PC2 explained 48.0% and 18.5% of the total variance, respectively, together accounting for approximately 66.5%. Single-factor PERMANOVA identified an association between species and the multivariate sequencing and assembly metrics (*p* = 0.001, *R*^2^ = 0.300). However, betadisper detected differences in dispersion among species (*p* = 0.002), so the PERMANOVA result may reflect differences in both group location and dispersion.

**Figure 4:**
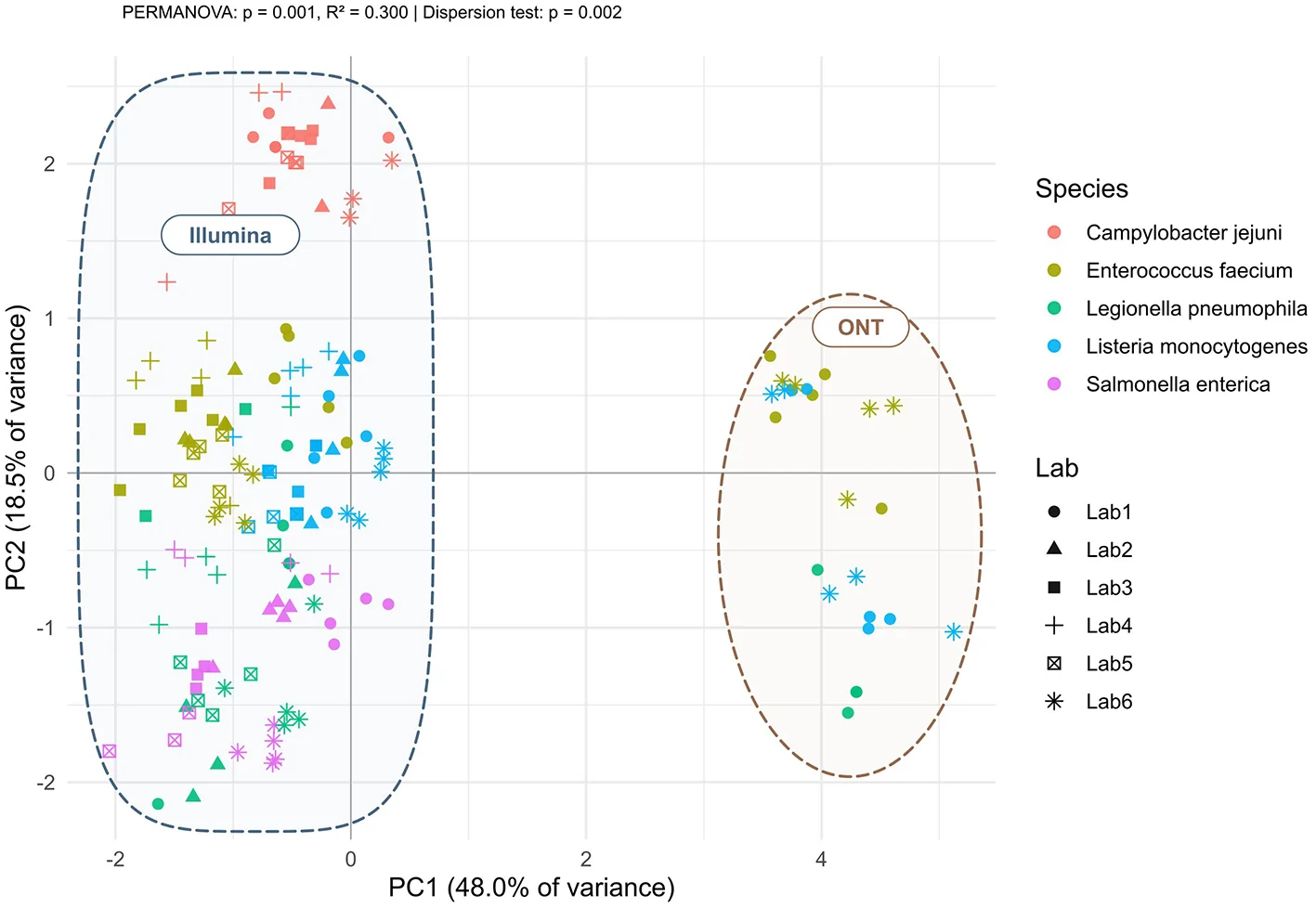
Principal component analysis of sequencing and assembly metrics after combined filtering. Each point represents a sequencing record from the pooled Illumina and Nanopore dataset. Colors indicate bacterial species and symbols indicate laboratory. The first two principal components explain 48.0% and 18.5% of variance, respectively. Species-based single-factor PERMANOVA and dispersion-test results are displayed.

To assess the sensitivity of factor contributions to filtering, marginal PERMANOVA *R*^2^ values were compared across four conditions: records meeting the minimum inclusion criteria without additional filtering, Q30 and contig-count filtering, PCA-based outlier filtering, and combined filtering. Species had the largest marginal R^2^ among the estimable explanatory factors in all three datasets and across all filtering conditions (Figure 5).

**Figure 5:**
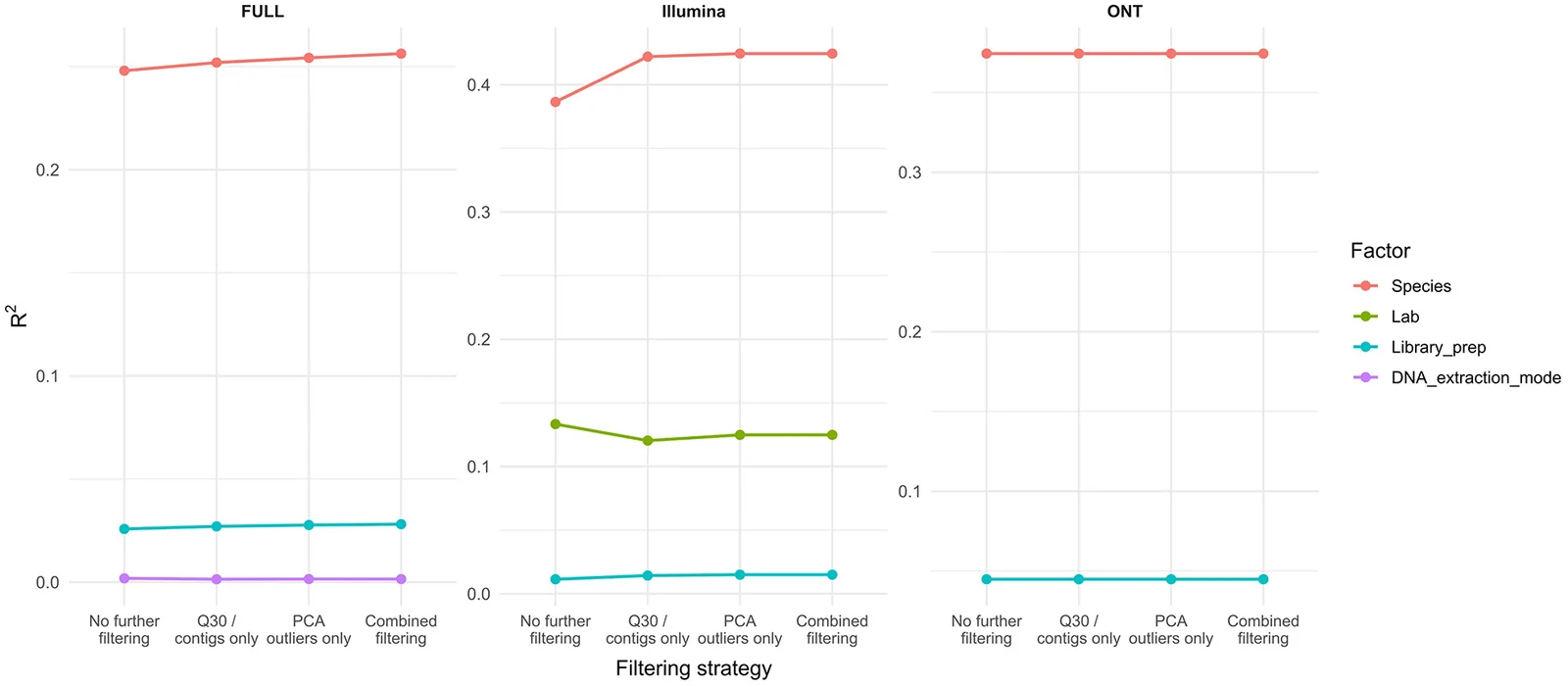
Sensitivity of marginal PERMANOVA effect sizes to filtering strategy. Lines show marginal R^2^ values for estimable explanatory factors in the pooled, Illumina and Nanopore datasets. All records met the predefined minimum inclusion criteria. Four conditions are compared: no further filtering, Q30 and contig-count filtering only, PCA-based outlier filtering only, and combined filtering, in which Q30 and contig-count filtering preceded PCA-based outlier removal. Vertical-axis scales differ between panels.

In the pooled dataset, the marginal R^2^ for species increased slightly from 24.8% without additional filtering to 25.6% after combined filtering. In the Illumina subset, it increased from 38.6% to 42.5%, whereas in the ONT subset it remained unchanged at 37.4% across all four filtering conditions.

The estimable technical factors had smaller marginal *R*^2^ values and showed limited variation across filtering conditions. In the Illumina subset, laboratory *R*^2^ ranged from 12.0% to 13.3%, while library preparation *R*^2^ ranged from 1.1% to 1.5%. Library preparation *R*^2^ ranged from 2.6% to 2.8% in the pooled dataset and remained at 4.5% in the ONT subset. DNA extraction mode accounted for approximately 0.1–0.2% in the pooled dataset. Other technical terms were not independently estimable in the corresponding multifactor models.

### 3.4 Typing outcomes and cross-laboratory reproducibility

Among records retained after combined filtering, 21 showed one or more allelic differences relative to the corresponding Lab 1 Illumina comparator assembly, including 14 Illumina-derived and seven ONT-derived records. For Illumina-derived records, allelic distances ranged from 0–2 for *L. pneumophila*, 0–1 for *C. jejuni*, and 0–1 for *S. enterica*, while no allelic differences were observed for *E. faecium* or *L. monocytogenes*. Among retained ONT-derived records, no allelic differences were observed for *E. faecium*; allelic distances ranged from 0–1 for *L. pneumophila* and 0–8 for *L. monocytogenes*. ONT records with < 95% cgMLST good-target recovery, including *S. enterica* records with incomplete allele profiles, were excluded before additional filtering and were not included in the allelic-distance analysis.

Among retained records with available cluster-assignment results, assignments were concordant across laboratories for the same biological isolate. Cluster-assignment information was unavailable for one retained record. After combined filtering, the isolate-attributable ICC for allelic distance was approximately 0.09 in the pooled dataset, 0.43 in the Illumina subset, and 0.60 in the ONT subset (Figure 6). The ONT estimate was based on 24 records from 14 biological isolates sequenced by two laboratories.

**Figure 6:**
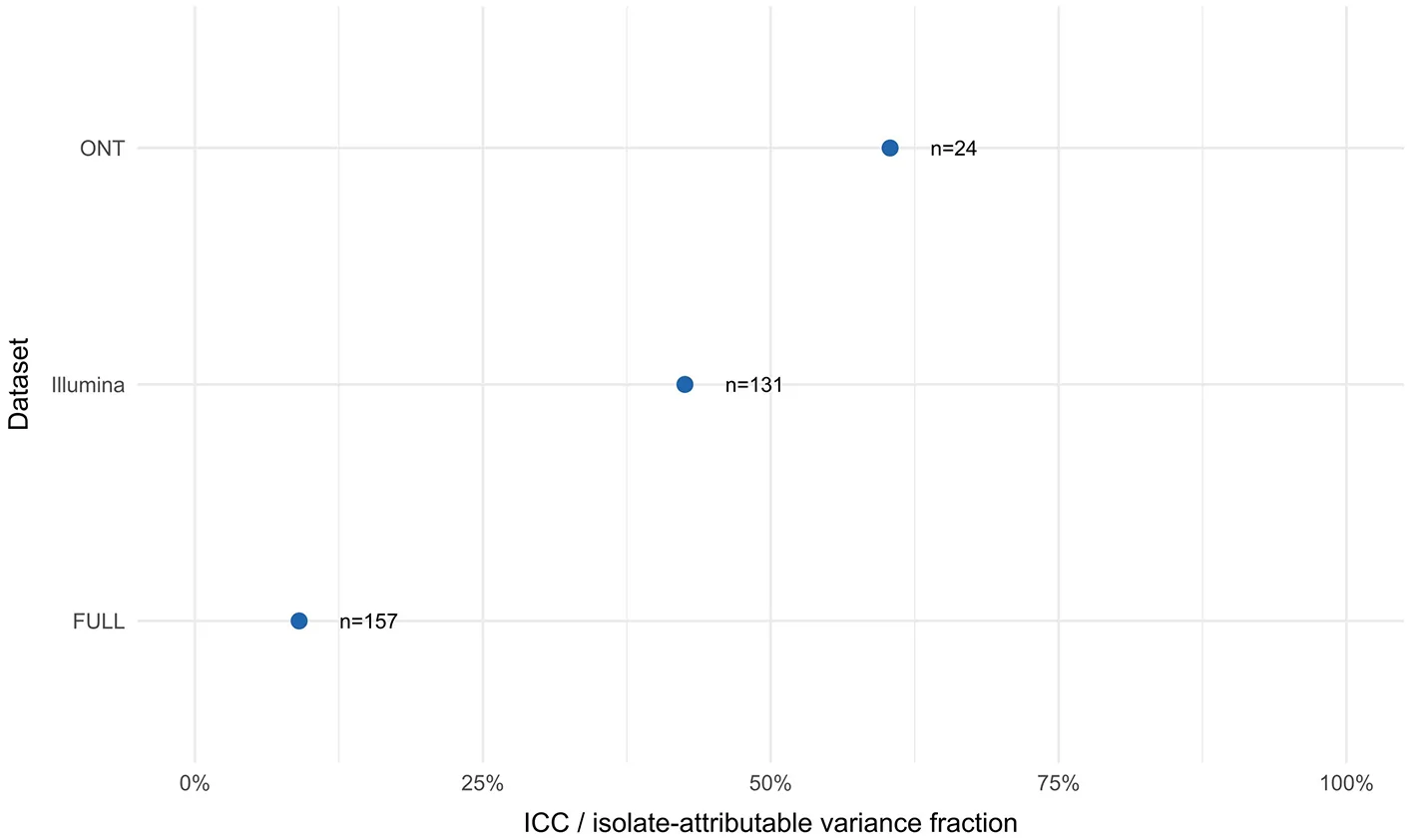
Isolate-attributable intraclass correlation coefficients for allelic distance after combined filtering. Points show ICC estimates for the pooled dataset and the Illumina and Nanopore subsets. ICC was calculated as biological-isolate variance divided by total variance from mixed-effects models with random intercepts for biological isolate and laboratory. Allelic distances were measured relative to the corresponding Lab 1 Illumina comparator assembly; labels indicate the number of sequencing records analyzed.

Bland–Altman analyses of the combined-filtered dataset showed small pairwise mean differences in allelic distance across Illumina laboratories, ranging from −0.24 to 0.15 alleles. Limits of agreement lay within −1 to +1 alleles for most comparisons. The Nanopore comparison involved 10 shared biological isolates and showed a mean difference of −0.50 alleles, with wider limits of agreement ranging from −3.60 to 2.60 alleles (Supplementary Figure 1).

In the combined-filtered dataset, Q30 base percentage showed low within-laboratory variability in both technology-specific subsets, with coefficients of variation ranging from 0.3% to 5.5% for Illumina and from 1.0% to 1.7% for Nanopore. Assembly metrics showed greater variability. In the Illumina subset, CVs ranged from 68.7% to 93.8% for N50 and from 40.2% to 68.3% for contig count. Corresponding ranges in the Nanopore subset were 7.2–11.7% and 27.0–35.7%, respectively (Supplementary Figure 2). These CVs summarize variation among records within each laboratory, reflecting both biological differences and technical variability; comparisons between technologies should therefore consider the smaller Nanopore subset and differences in isolate composition.

### 3.5 Mixed-effects

Mixed-effects variance partitioning in the combined-filtered dataset showed that the relative contributions of biological and technical factors differed across sequencing and assembly metrics (Figure 7). In the pooled dataset, sequencing technology accounted for 59.7% of the fitted variance in Q30 base percentage, followed by laboratory at 27.2%. Technology and assembler accounted for the largest components of N50 variation (47.4% and 40.4%, respectively) and contig-count variation (44.2% and 48.1%, respectively), whereas species accounted for 99.3% of assembly-size variation.

**Figure 7:**
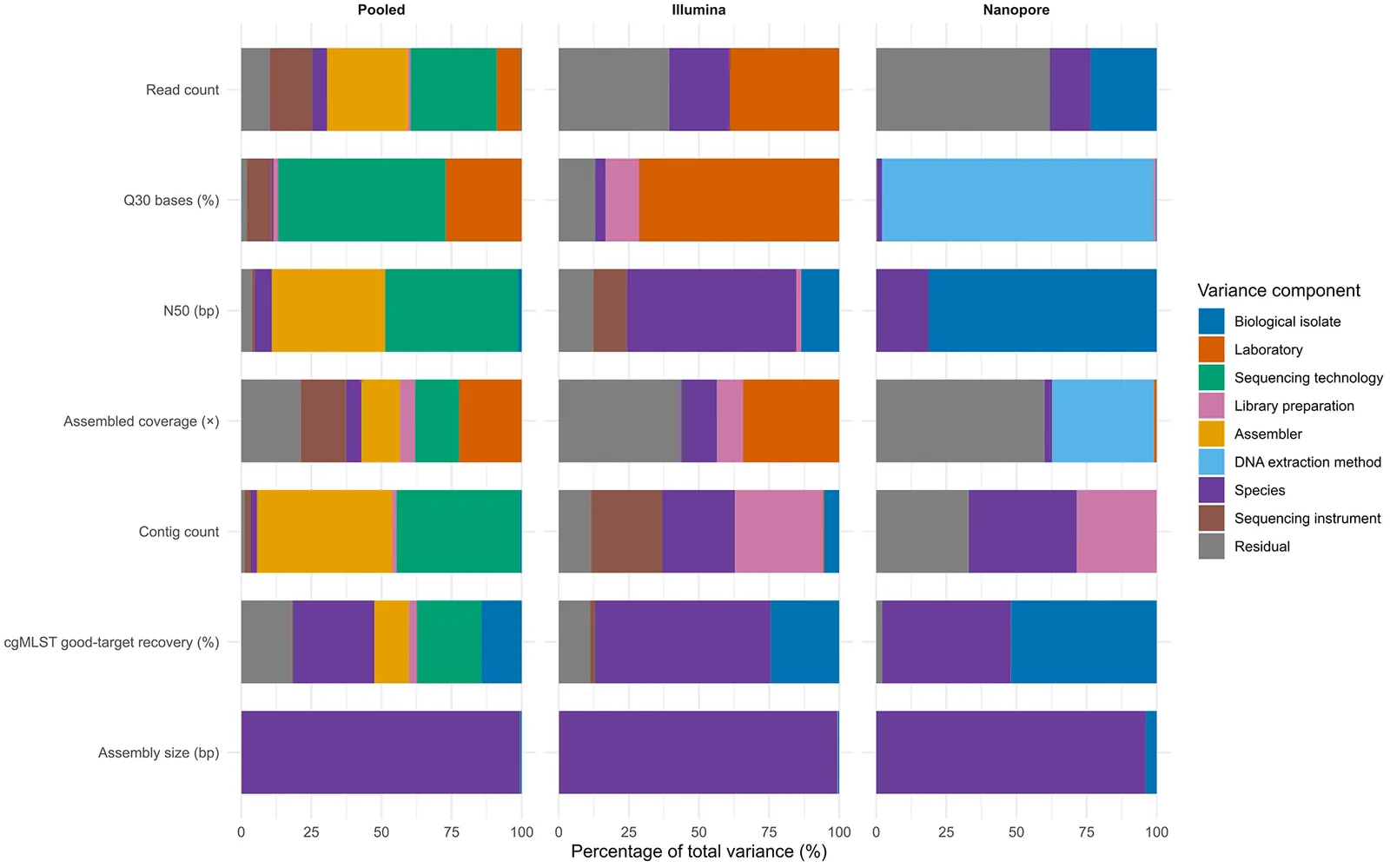
Mixed-effects variance partitioning of sequencing and assembly metrics after combined filtering. Stacked bars show the percentage of fitted total variance attributable to biological isolate, laboratory, sequencing technology, workflow factors, species and residual variation, where estimable. Results are presented separately for the pooled, Illumina and Nanopore datasets. Most models showed singular fits; variance components are therefore interpreted descriptively.

Within the Illumina subset, laboratory accounted for 71.3% of Q30 variation, while species accounted for 60.3% of N50 variation and 62.6% of cgMLST good-target recovery variation. Biological isolate identity contributed a further 13.5% and 24.5% to these latter two metrics, respectively. Contig-count variation was distributed mainly among library preparation (31.4%), species (25.9%), and sequencer (25.4%). In the Nanopore subset, biological isolate identity accounted for the largest variance components for N50 (81.3%) and cgMLST good-target recovery (52.0%).

Most models showed singular fits, with one or more variance components estimated as zero or near zero. These estimates were therefore interpreted as descriptive summaries of the fitted variance structure, with additional caution for the smaller Nanopore subset.

## 4 Discussion

Overall, our pilot ring trial demonstrated that harmonization of wet-laboratory workflows may not be necessary to achieve comparable cgMLST-based surveillance results across laboratories, provided that sequencing data meet predefined minimum quality requirements. Despite differences in sample handling, DNA extraction, library preparation, sequencing instruments, and local workflows, retained Illumina records showed only small allelic differences and concordant cluster assignments. These findings suggested that laboratories may retain established local workflows while generating comparable cgMLST results for surveillance if the resulting data are of sufficient quality for reliable typing.

Isolate recovery was generally high, although a small number of isolates could not be recovered, most notably *C. jejuni*. This is consistent with the known sensitivity of *C. jejuni* to environmental and handling conditions, which may induce a viable but non-culturable state (Jackson et al., 2009). Some sequencing records also failed the minimum coverage or cgMLST target-recovery requirements. This highlighted that flexibility in wet-laboratory workflows does not remove the need for appropriate quality criteria. The same principle is reflected in current European surveillance frameworks, which define acceptance criteria for WGS data rather than prescribing a single wet-laboratory workflow (Guerra et al., 2025).

As expected from the heterogeneous study design, sequencing and assembly characteristics varied across laboratories. Q30 showed clear laboratory-associated differences across species, whereas laboratory effects were less consistent for contig count and absent for N50. Multivariate analyses additionally showed, as expected, strong species- and technology-associated structure, with species remaining the largest estimable contribution across filtering strategies. The importance of species is consistent with the pathogen-specific approach used in European WGS proficiency testing. For example, the 2024 EURL-*Salmonella* exercise evaluated WGS-based cluster analysis using *Salmonella*-specific material across 24 laboratories (Jacobs-Reitsma et al., 2025). Overall, these analyses confirmed substantial biological and technical heterogeneity, but no single technical metric or workflow factor adequately described performance across all endpoints (Jacobs-Reitsma et al., 2025, 2026). Importantly, this heterogeneity did not translate into relevant differences in Illumina typing outcomes. Among retained records, cgMLST distances from the Lab 1 Illumina comparator were only 0-2 alleles, and cluster assignments were concordant across laboratories. Bland-Altman analyses similarly showed small differences among Illumina laboratories. Although the Illumina ICC was moderate, its interpretation is limited by the predominance of zero or near-zero allelic distances. For surveillance, absolute allelic agreement and concordant cluster assignment were therefore considered more informative than the ICC alone.

The observation that numerical differences did not necessarily change epidemiological interpretation is supported by recent work. Glasgow and coworkers compared three cgMLST pipelines across 255 isolates from eight bacterial species and found differences in allelic distances but very high concordance in cluster classification (Jacobs-Reitsma et al., 2025). Although that study examined analytical rather than wet-laboratory variation, both findings indicated that identical intermediate genomic measurements are not necessarily required for concordant epidemiological classification (Glasgow et al., 2025).

ONT results were less consistent and should be interpreted cautiously. Several ONT records, particularly for *S. enterica*, did not reach the required cgMLST target recovery. Because incomplete cgMLST profiles can affect allelic-distance estimates and consequently cluster assignment, records with < 95% good-target recovery were excluded from allelic-distance and clustering comparisons. These findings are consistent with reports of residual errors in ONT-only assemblies associated with basecalling, methylation, and homopolymeric regions (Bogaerts et al., 2025). Hybrid assemblies were deliberately not generated because the aim was to evaluate ONT alone. The results also reflected the technology available in 2023; ONT chemistry, basecalling, and error correction have since improved, and more recent studies indicate that ONT can approach Illumina performance for some pathogens (Bogaerts et al., 2025; Purushothaman et al., 2026). Current ONT workflows may therefore perform differently from those evaluated here.

Species-specific genomic characteristics may partly explain some of the observed variation. Genome size, GC content, repetitive sequences, mobile genetic elements, and homopolymeric tracts can affect sequencing coverage, assembly, and allele recovery (Treangen and Salzberg, 2011; Land et al., 2015; Merda et al., 2024; Qin et al., 2023), and recent European proficiency-testing programmes similarly evaluate WGS performance in a pathogen-specific context (Jacobs-Reitsma et al., 2025; European Centre for Disease, Prevention and Control, 2025). Sequence-composition bias associated with Nextera XT, for example, may be relevant for low-GC organisms such as *C. jejuni*. For *S. enterica*, several ONT records had incomplete cgMLST profiles despite high coverage and low contig counts; its larger genome, repetitive regions and mobile elements, combined with ONT-specific errors, may have affected locus recovery (Treangen and Salzberg, 2011; Land et al., 2015; Qin et al., 2023). However, because laboratory practices, library preparation, and sequencing instruments were partly confounded, these remain plausible explanations rather than causal effects demonstrated by this study (European Centre for Disease, Prevention and Control, 2025).

The additional statistical analyses supported rather than changed this overall interpretation. Coefficients of variation showed that Q30, N50, and contig count captured different components of variability, while variance partitioning identified different biological and technical contributions depending on the endpoint. No single wet-laboratory factor consistently dominated performance. Given the singular fits and confounding between several workflow variables, the mixed-effects results should be considered descriptive rather than causal.

Taken together, these findings support a pragmatic approach to multi-laboratory genomic surveillance, in which laboratories may retain validated local workflows provided that the resulting data are suitable for reliable typing. This is particularly timely in Europe, where WGS data-quality criteria and cgMLST-based data exchange are increasingly incorporated into surveillance and food-borne outbreak investigations. Our results show that such comparability does not necessarily require laboratories to use identical wet-laboratory workflows (Guerra et al., 2025).

This study has several limitations. The ONT subset was smaller than the Illumina dataset and included only two laboratories, and the ONT data reflect technology available in 2023. Only five isolates per species were included, limiting the genomic diversity represented. Several workflow factors were confounded because laboratories used their established procedures rather than a factorial study design, preventing attribution of variation to individual workflow components. Records that did not meet the predefined quality criteria were excluded from comparative analyses, although they were retained in the supplementary PASS/FAIL table. SNP-based analyses were not performed because the study focused on cgMLST. Finally, bioinformatic analyses were performed centrally using the same pipeline to specifically evaluate wet-laboratory heterogeneity. Whether similar concordance would be maintained across different analytical pipelines was outside the scope of this study, although the findings of Glasgow and coworkers suggest that some analytical heterogeneity may also be compatible with concordant cluster classification (Glasgow et al., 2025).

## 5 Conclusion

This pilot ring trial indicates that harmonization of wet-laboratory workflows may not be required for comparable Illumina-based cgMLST cluster interpretation, provided that sequencing data meet appropriate minimum quality requirements. Technical sequencing and assembly metrics varied across laboratories, but these differences did not prevent concordant cluster assignment. For multi-laboratory surveillance, ensuring that genomic data are fit for reliable typing may therefore be more important than requiring every laboratory to use the same wet-laboratory workflow. ONT findings were more species-dependent in this 2023 dataset and should be interpreted cautiously given the smaller ONT subset and the technology available at the time.

## Data Availability

The datasets presented in this study can be found in online repositories. The names of the repository/repositories and accession number(s) can be found in the article/Supplementary material.

## Supplementary material

Supplementary table 1 Metadata, sequencing quality metrics, assembly characteristics, and filtering outcomes for all sequencing records submitted to the SRA (BioProject PRJNA1484536) in the ring trial. PASS/FAIL columns indicate whether each record met the predefined minimum inclusion criteria and each additional filtering criterion. Records marked as FAIL for a given criterion were retained in the table for transparency but excluded from analyses requiring that criterion. Values marked as “n.a.” indicate information that was unavailable or not applicable for the corresponding record.

Supplementary figure 1 Bland–Altman comparisons of allelic distance between laboratories after combined filtering. Panels show 15 Illumina laboratory-pair comparisons and one Nanopore comparison. Points represent shared biological isolates, plotted by mean allelic distance and the difference between the first and second laboratory named in each panel. Solid blue lines indicate mean differences and dashed red lines indicate 95% limits of agreement. Labels indicate the number of paired isolates; panel scales vary.

Supplementary figure 2 Within-laboratory coefficients of variation for sequencing and assembly metrics after combined filtering. Bars show coefficients of variation for Q30 base percentage, N50 and contig count, separately for Illumina and Nanopore. CV was calculated as 100 × standard deviation/mean among records within each laboratory; values are labeled on the bars. Each metric has a common horizontal-axis scale across technologies. Variation includes both biological differences among isolates and technical variability.
